# Ssu72: a versatile protein with functions in transcription and beyond

**DOI:** 10.3389/fmolb.2024.1332878

**Published:** 2024-01-18

**Authors:** Emma Fidler, Katherine Dwyer, Athar Ansari

**Affiliations:** Department of Biological Sciences, Wayne State University, Detroit, MI, United States

**Keywords:** transcription, termination, CTD, gene regulation, gene looping, liver and autoimmune diseases

## Abstract

Eukaryotic transcription is a complex process involving a vast network of protein and RNA factors that influence gene expression. The main player in transcription is the RNA polymerase that synthesizes the RNA from the DNA template. RNA polymerase II (RNAPII) transcribes all protein coding genes and some noncoding RNAs in eukaryotic cells. The polymerase is aided by interacting partners that shuttle it along the gene for initiation, elongation and termination of transcription. One of the many factors that assist RNAPII in transcription of genes is Ssu72. It is a carboxy-terminal-domain (CTD)-phosphatase that plays pleiotropic roles in the transcription cycle. It is essential for cell viability in *Saccharomyces cerevisiae*, the organism in which it was discovered. The homologues of Ssu72 have been identified in humans, mice, plants, flies, and fungi thereby suggesting the evolutionarily conserved nature of the protein. Recent studies have implicated the factor beyond the confines of transcription in homeostasis and diseases.

## Discovery

The general transcription factors are required for initiation of transcription by RNAPII, the enzyme that transcribes the protein-coding genes (Woychik and Hampsey, 2002). Ssu72 was identified by the Michael Hampsey lab as an enhancer of a mutation in the general transcription factor TFIIB (Transcription Factor IIB) (Sun and Hampsey, 1996). The mutant was named *sua7-1.* Instead of simply compensating for the cold-sensitive growth defect of the mutant, the *ssu72-1* mutation conferred a temperature-sensitive phenotype in combination with the *sua7-1* mutation (Sun and Hampsey, 1996). In addition, the *ssu72-1* mutation enhanced the transcription start site defect of the *sua7-1* mutant (Wu et al., 1999; Pappas and Hampsey, 2000). Upon sequencing, *SSU72* was found to be a novel gene of unknown function that was essential for cell viability (Sun and Hampsey, 1996). Initial sequence analysis of Ssu72 did not reveal any structural features commonly associated with transcription factors, except for a clustering of acidic residues in the carboxy-terminal region of the protein. The allele specific interaction of Ssu72 with TFIIB as well as physical interaction of recombinant Ssu72 and TFIIB proteins *in vitro* suggested that Ssu72 must be playing a role in transcription by RNAPII (Sun and Hampsey, 1996; Wu et al., 1999).

The sequence of Ssu72 exhibited a limited similarity to ATP-dependent RNA helicases, which are enzymes that unwind RNA from an RNA-DNA duplex (Sloan and Bohnsack, 2018). There was, however, neither a motif for RNA binding nor for ATP-binding or ATP-hydrolysis in the primary structure of protein. This ruled out the possibility of Ssu72 being an ATP-dependent RNA helicase. The *ssu72-1* allele has a 30 bp duplication of a cysteine-rich region in the amino-terminal half of protein. This region exhibited similarity to the zinc binding motif (Pace and Weerapana, 2014). Site-directed mutagenesis revealed that a cysteine residue, cysteine-15, in the amino terminal of the protein is critical for its function. The CX_5_RS (C = cysteine, X = any residue, R = arginine and S = serine) sequence, which included cysteine-15, was identified as a protein tyrosine phosphatase motif (Ganem et al., 2003; Meinhart et al., 2003). Protein phosphatases are enzymes that remove phosphate group from proteins (Cohen, 1989). These observations culminated in the discovery that Ssu72 is a phosphatase specific for serine-5 of the carboxy-terminal-domain (CTD) of RNAPII (Krishnamurthy et al., 2004). The CTD is the carboxy-terminal domain of the largest subunit of RNAPII, Rpb1 (Phatnani and Greenleaf, 2006). Multiple laboratories identified Ssu72 as a subunit of the cleavage and polyadenylation factor (CPF) complex, a multi-protein complex that is involved in 3’ end processing of mRNA as well as termination of transcription (Dichtl et al., 2002; Gavin et al., 2002; He et al., 2003; Nedea et al., 2003) thereby implicating the protein in cleavage-polyadenylation and termination of transcription. TFIIB-Ssu72 interaction demonstrated for the first time the crosstalk of promoter and terminator ends of a gene during transcription (Al-Husini et al., 2020). How Ssu72 affects transcription start site selection, however, still remains unclear.

## Evolutionary implications

Although Ssu72 was discovered in budding yeast, similar proteins have been reported in multiple eukaryotes on the basis of sequence homology (Pappas and Hampsey, 2000; St-Pierre et al., 2005; Liu et al., 2021). Orthologs of Ssu72 have been identified in mammals including humans and mice, fungi like *Schizosaccharomyces pombe*, *Kluyveromyces lactis*, *Aspergillus flavus* and *Cryptococcus neoformans*, insects like *Drosophila melanogaster*, plants like *Arabidopsis thaliana* and Caenorhabditis *elegans* to name a few (Pappas and Hampsey, 2000; Chen et al., 2015; Park et al., 2023). Apart from the functional Ssu72 gene, several non-functional pseudogenes of the protein have also been identified in human and mice genomes (St-Pierre et al., 2005). A comparison of the primary structure of Ssu72 from different organisms revealed a 43% homology between yeast and human proteins and nearly 60% similarity between the fly and human counterparts (Pappas and Hampsey, 2000). The evolutionarily conserved nature of the protein emphasizes its functional significance across eukaryotic taxa, yet small variations in the structural makeup of the protein may have implications for the role it plays in different eukaryotes.

An investigation into the biological functions of Ssu72 in different eukaryotes revealed its role in both transcription and non-transcription related functions. The selective pressures governing the prevalence of Ssu72 across species may vary as the protein exhibits some species-specific phenotypes. In humans, it was discovered as a factor that interacts with the retinoblastoma (RB) tumor suppressor protein in a two-hybrid screen (St-Pierre et al., 2005). Although human Ssu72 is similar in primary structure to its yeast homolog and was able to physically interact with both human and yeast TFIIB, it was unable to complement the Ssu72 deletion mutation in budding yeast. These observations suggest that Ssu72 in humans may be involved in functions beyond the realms of transcription. The non-transcription functions of Ssu72 have also been reported in fungi *Aspergillus flavus* and *Cryptococcus neoformans*, where the protein was found to be involved in growth, development, pathogenicity as well as aflatoxin production (Rodríguez-Torres et al., 2013; Jin et al., 2020; Yang et al., 2020; Cossa et al., 2021). There are at least three plausible explanations for the multiplicity of Ssu72 functions among different eukaryotes. First, the primary role of Ssu72 is in transcription whereas non-transcription functions of the protein are indirect manifestations of its role in transcription. Second, the Ssu72 phosphatase picked up additional substrates during the course of evolution leading to multiplicity of non-transcription related functions. Third, sequence divergence of Ssu72 during evolution resulted in new interacting partners leading to its involvement in different non-transcription related processes in different eukaryotes. The implication of the protein in diverse cellular functions underscores the evolutionary retention of the protein in a wide range of eukaryotes.

## Biological functions

### Transcription-related functions

The transcription-dependent relationship between TFIIB and Ssu72 culminated in a rather unexpected discovery of the involvement of the factor at the 3′ end of genes. Analysis of the affinity purified CPF complex by mass spectrometry, an approach that identifies a protein on the basis of its amino acid composition, revealed Ssu72 as an integral component of the 3′ end processing complex (Dichtl et al., 2002; Gavin et al., 2002; He et al., 2003). Indeed, Ssu72 was found to play an important role in the 3′ end processing of both mRNA and snoRNA (Ganem et al., 2003; He et al., 2003; Steinmetz and Brow, 2003). It interacts strongly with Pta1, another subunit of the CPF complex responsible for pre-mRNA cleavage, polyadenylation, and termination (He et al., 2003). Ssu72 and Pta1 work together in the CPF complex, as shown by their interaction and the correlating balanced levels of each protein (He et al., 2003). It was also found that Ssu72 has a novel role in termination of transcription of at least a subset of genes (Ganem et al., 2003). Ssu72 also facilitates the termination of transcription for non-coding RNA like snRNA or snoRNA in yeast (Wani et al., 2014). The role of the phosphatase activity in snRNA termination is not limited to yeast. In fact, in a mammalian cell line it was demonstrated that upon depletion of Ssu72, the 3′ end formation of U2 and U4 snRNA is defective (Wani et al., 2014). Unlike mRNA termination, where the CPSF complex is involved, snRNA termination in humans is regulated by the Integrator complex. Ssu72 associates with the Integrator complex in humans to promote efficient 3′ end processing and termination of transcription, showcasing its versatile role in the transcription process (O’Reilly et al., 2014). Thus, the role of Ssu72 in 3′ end processing and termination has been conserved during evolution (Steinmetz and Brow, 2003; Wani et al., 2014).

X-ray diffraction analysis, an approach that reveals the high resolution three-dimensional structure of proteins, DNA and other molecules, revealed a conserved catalytic core with the characteristic phosphatase motifs. The catalytic domain consists of a signature aspartic acid residue within the active site, which is characteristic of metal-dependent phosphoprotein phosphatases (Xiang et al., 2010; Zhang et al., 2011). The phosphatase activity of Ssu72 was confirmed using a synthetic substrate (Ganem et al., 2003). The presence of phosphatase motifs, validation of phosphatase activity *in vitro* and genetic interaction with Kin28 and Ctk1 CTD kinases as well as Fcp1 CTD phosphatase strongly suggested that Ssu72 might be a CTD phosphatase. This hypothesis was confirmed when it was demonstrated that Ssu72 has a phosphatase activity specific for serine5p (phosphorylated serine5) of CTD in budding yeast (Krishnamurthy et al., 2004). Initial analyses found that Ssu72 has serine5p phosphatase activity only. Further investigation, however, revealed it to have serine7p (phosphorylated serine7) phosphatase activity as well (Bataille et al., 2012). The substrate specificity of Ssu72 for serine5p or serine7p is dependent on the orientation of Ssu72 relative to the backbone polarity of the CTD. In one orientation, Ssu72 specifically dephosphorylates serine5p, whereas in the opposite orientation, it targets serine7p (Xiang et al., 2012). The phosphatase activity was found to regulate the transcriptional state of RNAPII by altering its phosphorylation state during different steps of transcription (Dichtl et al., 2002; Rosado-Lugo and Hampsey, 2014). The role of Ssu72 phosphatase activity during the transcription cycle has been highly conserved across eukaryotic taxa.

**FIGURE 1:**
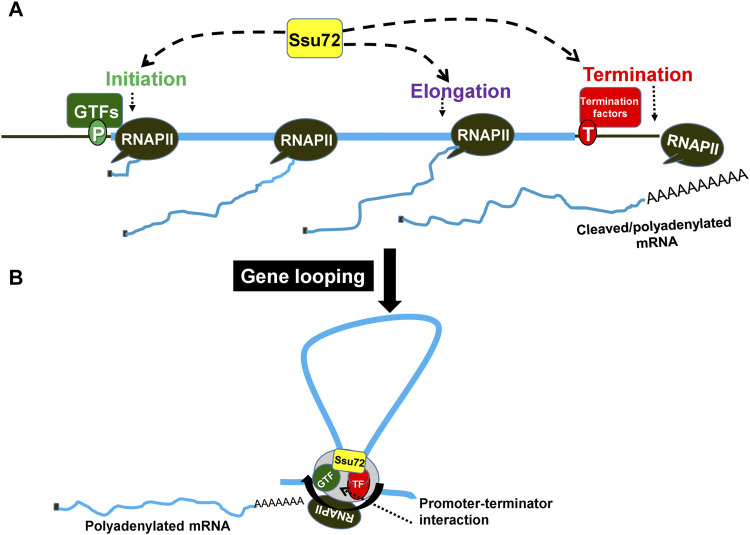
Ssu72 facilitates a gene looped formation bridging the promoter and terminator regions of a gene. **(A)** Ssu72 is involved in initiation, elongation and termination of transcription. **(B)** During transcription, the gene adopts a looped conformation wherein the promoter and terminator physically interact with each other. This interaction is beneficial for reinitiation and is guided by the association of termination factors with TFIIB. GTFs, general transcription factors; TF, termination factors.

An analysis of the genetic and physical interactions of Ssu72 with the promoter-bound factors implicated the protein in the assembly of the preinitiation complex (PIC) (Ganem et al., 2003; Spector et al., 2019). Prior to initiation of transcription, the preinitiation complex (PIC) recruits RNAPII with a hypo-phosphorylated CTD. Ssu72 associates with RNAPII during formation of the PIC, specifically interacting with the Rpb2 and Rpb4/7 subunits (Pappas and Hampsey, 2000; Allepuz-Fuster et al., 2014). During the preinitiation stage, Ssu72 targets its phosphatase activity on the CTD (Spector et al., 2019). It facilitates assembly of the PIC by keeping the CTD unphosphorylated until transcription is started (Spector et al., 2019). Following initiation of transcription, serine5 of the CTD is phosphorylated by Kin28 or CDK7, a subunit of TFIIH. This phosphorylated serine5 (serine5p) promotes recruitment of more factors to open up the chromatin structure for transcription to take place and to cap the newly growing transcript. The serine5 phosphorylation is also required for the promoter clearance step, thereby allowing the polymerase to go into productive elongation. Once RNAPII escapes the promoter, Ssu72 regulates the transition to the elongation phase by incrementally dephosphorylating serine5p (Reyes-Reyes and Hampsey, 2007; Rosado-Lugo and Hampsey, 2014). Prior to termination, serine5p and serine7p are almost completely dephosphorylated by Ssu72, thereby priming the RNAPII for termination of transcription. (Reyes-Reyes and Hampsey, 2007; Zhang et al., 2012). Thus, Ssu72 directly or indirectly participates in almost every step of the transcription cycle from initiation to termination (Figure 1).

Ssu72 might be the factor that links termination to reinitiation of transcription (Figure 1). The interaction of Ssu72 with CPF termination complex, and the general transcription factor TFIIB revealed for the first time a novel link between the initiation and termination steps of transcription (Ansari and Hampsey, 2005; Singh and Hampsey, 2007; Bratkowski et al., 2018; Allepuz-Fuster et al., 2019). The promoter-terminator interaction results in formation of a looped gene architecture, where the distal ends of a gene come into proximity (O’Sullivan et al., 2004; Ansari and Hampsey, 2005; El Kaderi et al., 2009; Al-Husini et al., 2020). A consequence of this looped conformation is coupling of termination with reinitiation through facilitated transfer of polymerase from terminator to the promoter (Figure 1). Upon inactivation of Ssu72, the gene loop formation is disrupted (Ansari and Hampsey, 2005). This is most likely due to loss of interaction between Ssu72 and TFIIB (Medler et al., 2011). The crystal structure of the TFIIB-Ssu72 complex revealed that the negatively charged surface of the outer helix of Ssu72 binds to the positively charged surface of helix 5 of cyclin like domain C1 of TFIIB (Bratkowski et al., 2018). The second cyclin like domain C2 is required for full binding activity. Binding interface between Ssu72 and TFIIB is conserved across species. Ssu72 contains an overlapping yet non-identical binding surface for TFIIB carboxy-terminal domain and symplekin (Pta1), which is a subunit of CPSF (CPF) termination complex (Xiang et al., 2010). Structural studies suggest that Ssu72 facilitates reinitiation of transcription by handing off active TFIIB to the promoter in a gene loop (Bratkowski et al., 2018). Additionally, Ssu72 phosphatase activity is responsible for priming RNAPII for reinitiation through hypo-phosphorylation of the CTD. Gene looping occurs in an activator-dependent manner thereby suggesting that termination-reinitiation coupling may be crucial for activated transcription (El Kaderi et al., 2009).

### Transcription-independent functions

The repertoire of Ssu72 functions extends well beyond transcription. Novel roles of the protein have been discovered in fission yeast and higher eukaryotes. It has been implicated in telomere maintenance, chromatid cohesion and condensation, thermogenesis and phosphate homeostasis.

Ssu72 acts as a crucial regulator of phosphate homeostasis in fission yeast. It has been demonstrated that Ssu72 controls the phosphorylation states of serine5 and serine7 of the CTD of RNAPII (Schwer et al., 2015; Sanchez et al., 2018). Ssu72 serves as an intracellular sensor of the levels of phosphate within the cell and modulate expression of the Pho1 protein, a key regulator in the phosphate pathway (Sanchez et al., 2019; Sanchez et al., 2020). The homeostatic fate of phosphate in fission yeast is determined by the proper expression of Pho1. The phosphate regulatory axis is directly impacted by the RNAPII CTD phosphorylation status, which in turn dictates Pho1 expression pattern. Pho1 then regulates intracellular phosphate concentrations by facilitating the breakdown of phosphate. Notably, recent investigations reveal an intricate network of proteins involved in the phosphate homeostasis pathway that links CTD phosphorylation with transcriptional termination (Garg et al., 2022). These new findings therefore connect transcription termination with the regulation of specific gene expression in response to phosphate levels. Fluctuations within the phosphate signature of CTD by Ssu72 feeds back into maintaining the phosphate level in the cell thereby showcasing another unique role of Ssu72.

In fission yeast, the protein has been implicated in telomere replication. Specifically, it regulates the telomere replication cycle, generally referred to as telomere homeostasis (Escandell et al., 2019). Ssu72 activates lagging strand synthesis by facilitating the recruitment of Stn1 subunit of the Stn1-Ten1 complex with DNA polymerase α to the replicating telomeres (Escandell et al., 2019). DNA polymerase α synthesizes the primer to initiate DNA replication (Lehman and Kaguni, 1989). Ssu72 affects recruitment of Stn1 by regulating its phosphorylation status. Ssu72-mediated dephosphorylation of Stn1 facilitates its recruitment to telomeres, thereby affecting telomere length and stability. Human telomerase complex also exhibits a similar regulation by Ssu72.

In HeLa cells, Ssu72 was found to have a role in cohesion of sister chromatids as well as chromosome condensation (Kim et al., 2010). Cohesin is loaded onto the replicated sister chromatids during S-phase. Cohesin complex is composed of Smc1, Smc3, Rad21 and either SA1 or SA2 subunits. Ssu72 associates with the cohesin complex soon after its deposition during S-phase. Ssu72 prevents premature disassociation of cohesin from duplicated sister chromatids. Ssu72 does so by affecting the phosphorylation of cohesin subunit SA2 through its phosphatase activity. Consequently, when Ssu72 is inactivated, sister chromatids separate prematurely due to dissociation of cohesin complex before metaphase. Ssu72 also affects chromosome condensation during cell division (Bernard and Vanoosthuyse, 2015). The role in chromosome condensation is by virtue of it being a component of CPF complex (Vanoosthuyse et al., 2014). Within the CPF complex, there is a subcomplex called DPS complex consisting of Dis2, a WD-repeat protein Swd2.2, and a putative phosphatase regulatory factor Ppn1. This DPS complex associates with the chromosomes during early mitosis and facilitates recruitment of condensins on chromosomes (Vanoosthuyse et al., 2014). Dissociation of DPS complex from chromosome, however, is essential for condensation of chromosomes and therefore for progression of cell cycle. Ssu72 and DPS complex play redundant roles in chromosome condensation. Like DPS complex, Ssu72 is required for recruitment of condensins in early mitosis, but must dissociate to allow condensation (Vanoosthuyse et al., 2014). Ssu72, therefore, a negative regulator of chromosome condensation upon entry of cell in mitosis. It is believed that Ssu72 phosphatase activity is involved in the process. The target of the phosphatase, however, has not yet been identified.

Another non-transcriptional role of Ssu72 is in thermogenesis in mammals. Brown adipose tissue (BAT) is the specialized fat within the human body that is rich in mitochondria and is required for thermogenesis. The phosphatase activity of Ssu72 has been implicated in conferring cold tolerance by affecting the physiology of brown adipose tissue (Lee et al., 2018). In fact, Ssu72 expression is significantly higher in brown adipose tissue as compared to other tissues, indicative of its involvement in BAT function. The protein has been found to affect translation in brown adipose tissue by targeting the translation initiation factor eIF2α. Ssu72 regulates translation initiation on a global scale in BAT. Upon downregulation of Ssu72, a severe mitochondrial dysfunction phenotype occurs (Park et al., 2023). Consequently, depletion of Ssu72 in the BAT of mice leads to abnormal thermoregulation (Lee et al., 2018). These findings stipulate that Ssu72 is essential for thermogenic adaptation in mammals.

Ssu72 functions outside of transcription also encompass its unique role in regulating alveolar macrophage (AM) development and allergic airway inflammation. Ssu72 phosphatase activity was found instrumental in regulating the development, function and maturation of AMs through binding to the GM-CSF (Granulocyte Macrophage-Colony Stimulating Factor) receptor and influencing it is signaling (Woo et al., 2021). GM-CSF receptor is an essential membrane receptor responsible for cytokine production, antigen uptake, mitochondrial function and MHCII (Major Histocompatibility Complex II) expression. GM-CSF is the target of Ssu72 phosphatase. In the absence of Ssu72, GM-CSF signaling is dysregulated thereby affecting the function of AMs and attenuating an allergic airway inflammation response (Woo et al., 2021).

### Disease related aspects

Multiplicity of Ssu72 functions is manifested in its involvement in several human diseases. The protein has been implicated in liver function, hepatocellular carcinoma (HCC), immune system regulation, autoimmune diseases, and HIV.

#### Ssu72 in liver pathogenesis

**FIGURE 2:**
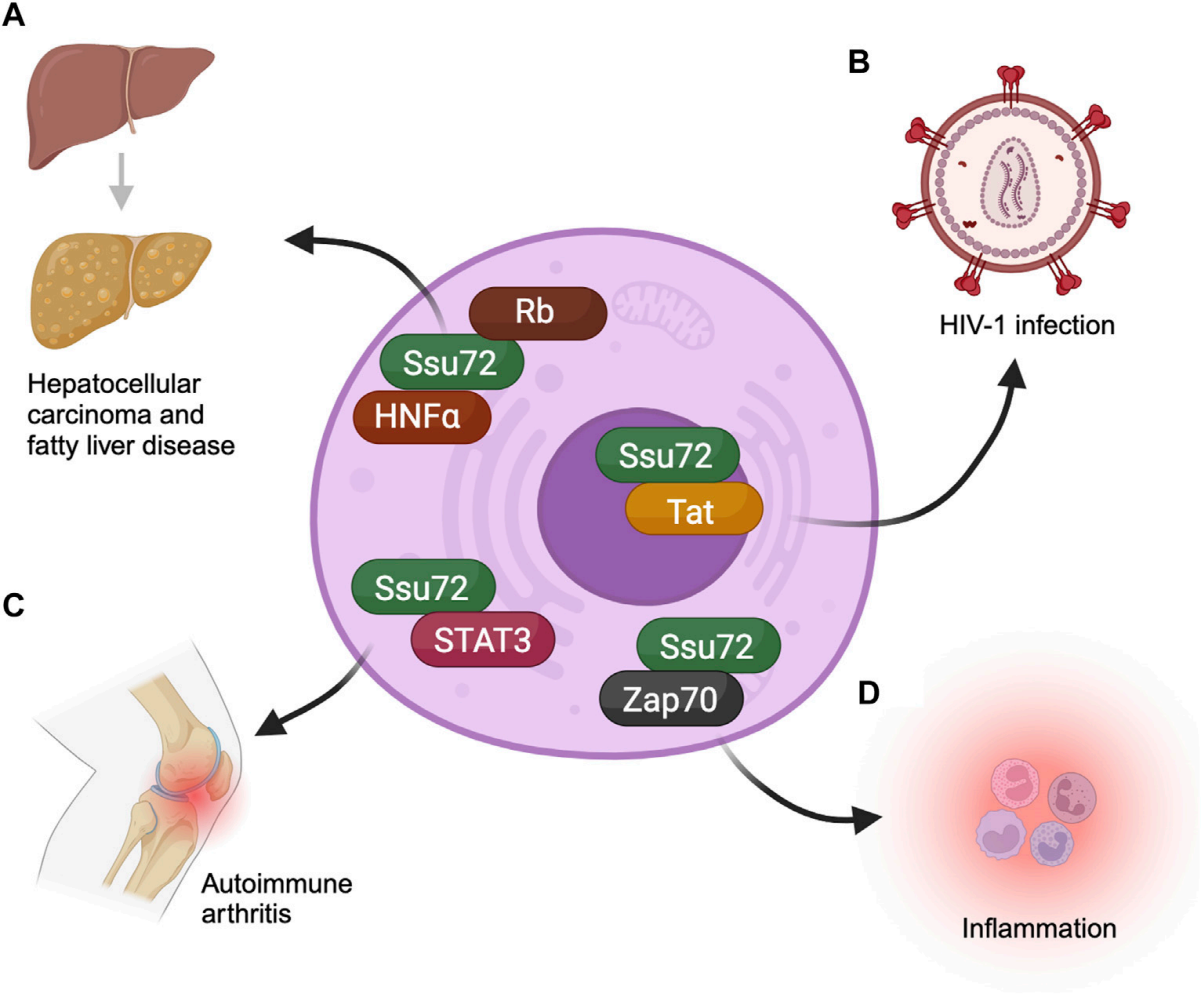
Ssu72 is involved in multiple disease-related pathways. **(A)** Ssu72 interacts with HNFα and Rb to regulate hepatocyte differentiation. However, upon depletion of Ssu72 the differentiation is disrupted, leading to conditions such as hepatocellular carcinoma and fatty liver disease. **(B)** Ssu72 associates with HIV-1 transcriptional activator Tat, promoting transcription of HIV-1 in host cells. **(C)** It also plays a critical role in the inflammation pathway connected with the autoimmune collagen-induced arthritis by dephosphorylating a key regulator, STAT3. Overexpression of Ssu72 inhibits inflammation. **(D)** ZAP70, a crucial regulator of T-cell differentiation, associates with Ssu72 thereby modulating the phosphorylation status of the protein to prevent spontaneous inflammation.

Hepatocytes are cells that make up approximately 70% of the liver. They are unique in that they are often polyploid (Gupta, 2000). The ploidy level as well as the number of nuclei in hepatocytes is developmentally regulated (Epstein, 1967). The higher ploidy level serves as a defense mechanism against high stress. A disruption in the relative proportion of the polyploid and diploid hepatocytes has been observed in damaged liver tissue (Gupta, 2000). It has been found that ploidy level of hepatocytes is regulated by Ssu72. Deletion of Ssu72 from mice hepatocytes resulted in mononuclear condition and aberrant ploidy level (Kim et al., 2016). Mechanistically, Ssu72 dephosphorylates Rb protein (Kim et al., 2016). Rb is a tumor suppressor and a cell cycle regulator (Weinberg, 1995). Increased phosphorylation of Rb in the absence of Ssu72 adversely affects its ability to regulate cell cycle checkpoint. The net result is hyper replication of DNA and consequent increase in the ploidy level of liver cells leading to liver damage (Kim et al., 2016). Thus, the role of Ssu72 in maintaining the ploidy level and nuclear number in hepatocytes is crucial for normal liver functions (Figure 2).

Inactivation of Ssu72 is also linked to hepatocellular carcinoma (HCC) (Kim et al., 2022). HCC is a common form of liver cancer that results from chronic liver damage. The inactivation of Ssu72 affects the probability of developing HCC. Ssu72 levels are significantly lower in hepatocytes of individuals with HCC compared to healthy individuals (Kim et al., 2022). Hepatic carcinoma may be due to dysregulation of Rb in liver cells in the absence of Ssu72. There is, however, evidence that Ssu72 also regulates the transcription factor Hepatocyte nuclear factor-4 (HNFα), which plays a key role in hepatocyte differentiation (Kim et al., 2022). The transcription function of HNFα is regulated by phosphorylation (Guo et al., 2003). When Ssu72 is depleted, HNFα undergoes hyperphosphorylation and its transcription function is compromised. Consequently, HNFα regulated genes are downregulated leading to aberrant proliferation and dedifferentiation of hepatocytes (Kim et al., 2022). The dedifferentiation of hepatocytes mark their transition to tumor progenitor cells. These results suggest a connection between Ssu72 phosphatase activity and hepatic carcinoma (Figure 2).

#### Ssu72 and immune response

The adaptive immune system is made up of T lymphocytes that produce lymphokines, and B lymphocytes that produce antibodies (Cooper and Alder, 2006). T cells can differentiate into helper T cells, regulatory T cells, or cytotoxic T cells. The balance between T cells and B cells and their subcategories is essential for proper immune response. When Ssu72 is depleted, differentiation into helper T cells is elevated while differentiation into regulatory T cells is reduced (Ko et al., 2021; Lee et al., 2021). These observations implicate Ssu72 in fine-tuning T cell receptor (TCR) signaling. The TCR signaling pathway is a complex cascade of molecular events that occurs when a T cell recognizes and binds to an antigen (Huse, 2009). In the TCR signaling pathway, a balance of phosphorylated and unphosphorylated target proteins is necessary for proper propagation of the signal (Courtney et al., 2018; Hwang et al., 2020). It was proposed that Ssu72 may regulate this phosphorylation-dephosphorylation balance through its phosphatase activity. The hypothesis gained credence when Ssu72 was identified as a modulator of phosphorylation of the TCR signaling protein ZAP70 (Ko et al., 2021). ZAP70 is required for initiation of TCR signaling, and its hyperphosphorylation in the absence of Ssu72 results in over activation of the pathway, leading to spontaneous inflammation (Ko et al., 2021). The overall conclusion of these observations is that Ssu72 is required for proper T cell differentiation and inflammation response due to its ability to regulate TCR signaling pathway through its phosphatase activity (Figure 2).

Autoimmune diseases refer to systemic or tissue-specific diseases caused by the immune system attacking the normal body cells (Davidson and Diamond, 2001). When the immune response is compromised, it can result in misrecognition of individual’s own cells as foreign cells. Ssu72 affects the autoimmune response by negatively regulating signaling cascades in the immune cells through its phosphatase activity (Kim and Lee, 2023). Ssu72 is also involved in dephosphorylation of the transcription factor STAT3 (signal transducer and activator of transcription-3), which is involved in Th17 cell differentiation (Lee et al., 2017). Th17 cells are a subset of helper T cells that produce interleukin-17 (Afzali et al., 2010). It was found that the overexpression of Ssu72 in Th17 cells can inhibit inflammation pathways by dephosphorylation of STAT3 (Lee et al., 2017) (Figure 2). In a mouse model, this overexpression exerted a therapeutic effect on autoimmune collagen-induced arthritis, implicating Ssu72 as a possible target for treatment of the disease (Lee et al., 2017).

#### Ssu72 and HIV

Ssu72 also plays a role in the transcription of the human immunodeficiency virus-1 (HIV-1) in host cells (Figure 2). The HIV-1 highjacks the host gene expression machinery in order to propagate itself. HIV-1 transcriptional activator Tat represses the antiviral genes of the host, while stimulating the expression of its own genes (Liang and Wainberg, 2002). HIV-1 Tat requires Ssu72 and its CTD phosphatase activity to promote transcription of viral genes (Chen et al., 2014). It was found that HIV-1 Tat directly interacts with the carboxy-terminal end of Ssu72 to stimulate its CTD phosphatase activity, in a manner similar to the human symplekin termination factor (Chen et al., 2014). Ssu72 knockdown showed an increase in serine5-CTD phosphorylation during transcription of HIV-1. Serine5 phosphorylation of CTD is carefully regulated during transcription. This increase in serine5-CTD phosphorylation in the absence of Ssu72 is associated with a decrease in transcriptional efficiency. However, the knockdown had no effect on global serine5p-CTD levels. In humans, Ssu72 was found dispensable for transcription at most cellular genes, making it a potential valuable target for selective disruption of viral transcription (St-Pierre et al., 2005).

## Conclusion

Ssu72 plays multifaceted roles in eukaryotic biology, with significant functions within and outside the realm of transcription. Although Ssu72 was discovered as a factor that genetically and physically interacts with the transcription initiation factor TFIIB, it turned out to be an evolutionarily conserved protein with multiple roles in transcription as well as non-transcription related processes. Within the transcription cycle, Ssu72 affects initiation, termination, termination-coupled reinitiation as well as crosstalk of promoter with the terminator region. Outside the transcription cycle, Ssu72 has been implicated in chromatid cohesion and condensation, regulation of ploidy level, telomere replication, thermogenesis, phosphate homeostasis, immune response and liver functions. Whether the effect of Ssu72 in non-transcription related functions is a manifestation of its role in transcription, or consequence of its newly acquired phosphatase substrates, or new interacting partners is an avenue for further exploration.

Involvement of Ssu72 in multiple human pathogenic conditions makes it a potential target for finding a cure for these diseases. An investigation into the mechanisms underlying involvement of the protein in affecting ploidy levels and differentiation in hepatocytes could provide an opportunity for understanding hepatic carcinoma in humans. Uncovering the possible therapeutic implications of targeting Ssu72 in autoimmune disease merits further exploration as well. Since Ssu72 is not required for transcription of all host genes, but is crucial for HIV-1 propagation, it is a potential target for finding an efficient treatment for the viral disease. Investigation into the role of Ssu72 in thermogenic tolerance may pave the way for discovery of a treatment for metabolic diseases like obesity related diseases and non-alcoholic fatty liver disease. The multifunctional roles of Ssu72 and its relevance in normal cellular and disease-related processes makes the protein an attractive subject for future research with wide ranging implications in biology and medicine.
